# ‘Not My Child’: Parents’ Denial About Adolescent Sexuality in Harare, Zimbabwe 

**Published:** 2017-09

**Authors:** Manase Chiweshe, Malvern Chiweshe

**Affiliations:** 1 Institution of Lifelong Learning, Chinhoyi University of Technology, Chinhoyi, Zimbabwe; 2 Critical Studies in Sexualities and Reproduction, Rhodes University, Grahamstown, South Africa

**Keywords:** Adolescent, Sexuality, Denial, Sexual Scripts

## Abstract

**Objective:** To find out adult views on adolescent sexualities in Zimbabwe and how adults construct sexual cultures that deny adolescence access to sex.

**Materials and methods:** The paper uses qualitative methodologies, with purposively selected parents and key informants. A total of ten in depth interviews, four focus groups and six key informant interviews with purposively sampled male and female respondents were conducted. Key informants included a headmaster, teacher, social worker, nurses and a member of traditional healers association.

**Results:** Parents that were interviewed denied that their adolescent children were sexually active. This denial of adolescent sexuality was seen throughout the interviews. The denial of adolescent sexuality was linked to the other themes that emerged including sexual surveillance and sexual communication, school pregnancy, STIs and sexual education, and adult anxiety on adolescent sex.

**Conclusion:** The denial of youth sexuality has serious impacts on youths’ access to information and ability to protect themselves from sexually transmitted diseases and HIV. We argue that government policies and lack of comprehensive sex education in schools are based on this denial of adolescent sexuality and should be addressed.

## Introduction

There is widespread believe in Zimbabwe based on cultural and religious norms, that sex is for adults (1). Young people are expected to avoid sexual contact of any kind as this is seen as immoral (2). Dominant sexual scripts deny adolescent sexual agency and confirm adult control of youth sexuality (1). These sexual scripts have permeated through to the education system where no comprehensive sexuality education curriculum exists. Parents have been at the centre of refusing such programmes to be taught in schools as this encourages children to be promiscuous (3). This paper outlines the thoughts, beliefs and views of parents and the elderly on sexual activity amongst adolescents in Zimbabwe. It uses Mbare high-density suburb located in Harare, the capital of Zimbabwe. Through qualitative methodologies, the paper weaves an intricate narrative of how parents refuse to accept that their children are sexually active. This denial is the basis of parents’ lack of involvement in sexual issues that affect their children. In the era of HIV and AIDS parents cannot afford to ignore sex as a critical part of their children’s life. Parents in particular play a substantial role in the gender and sexual socialization of their

children (4). It is thus interesting to analyse how and why parents construct views on adolescent sexuality. One fifth of the world’s population (a total of 1.2 billion people) are adolescents, and 85% of them are in the developing world (5). As such this age group is important for the future especially in Africa. Research on adolescence has suggested that this age group is at serious risk of HIV and AIDS, pregnancies and sexual transmitted diseases due to unprotected heterosexual sex (5). Given that half of all HIV infections occur in young people, this risk is real (6). The World Health Organization defines adolescents as aged 10 to 19 years, an age span that overlaps with UN definitions of children (0 to 18 years) and youth (15 to 24 years) (5). This period includes different stages of physical, emotional, psychological, social and cognitive development. Critical theorists who see it as a social construct have challenged adolescence as a universal period of transition between childhood and adulthood (7). The period of adolescence is a Euro-centric, western concept that has been transported to Africa by historical processes such as colonialism and globalisation (7). 

Across Africa adult public opinion has shown that premarital sex is viewed as wrong and dangerous to health, resulting in abortions, teenage mothers and sexually transmitted infections (5, 8). In such African spaces the dominant discourses on sex are dominated by partriachial ideologies. These partriachial ideologies construct adolescence in ways that limit their sexual agency. Despite the elders (especially male) controlling sexuality, young people create spaces to express and practise their sexuality especially with the advent of social networks where adult control is limited. Research has shown that parent’s involvement in adolescent sexualities is influenced by how they view adolescence as a stage of development (7). Despite adolescence not being biological but rather socially, contextual, spatially and temporally relative, parents and other societal members see adolescence as a universal part of development. Historical processes such as colonialism and globalisation, which has transported their view of adolescence, have influenced Zimbabwe, like other African countries (7). As such the way adolescence is viewed is usually through western constructs. In Africa, the western views of adolescence are usually mixed with traditional and conservative views of what adolescence should be (asexual). In western contexts ‘turmoil and rebellion are seen as the hallmarks of adolescence (4). Adolescence is thus related to concepts such as *sturm und drang, *identity crisis, identity construction, youth rebellion and youth culture and resistance. People going through adolescence are then seen as transitioning from childhood to adulthood and thus should not be sexually active (7). Adolescence is often equated to childhood as young men and women are often treated as children. Due to the transitioning adolescences are seen as constantly needing the guidance of adults so that they do not ruin their lives by having unprotected sex amongst other social ills like drugs (7).

In analysing parent adolescent sexual communication, it has been noted that it tends to be authoritarian and uni-directional, characterized by vague warnings rather than direct, open discussion (9). This can be linked to how parents construct adolescence as discussed above. Parents avoid discussing sexuality ‘with their children because they may feel inhibited by cultural or personal taboos or lack accurate information themselves’ (3, 9). The issue of lack of information which has been also found in Zimbabwe extends to parents not having the ‘age-appropriate respectful vocabulary and skills’ to talk to their adolescents (3, 10, 11). While other parents have been seen to believe that ‘discussing sexual matters with adolescents can promote early sexual intercourse’ (9). These attitudes can prompt an “abstinence-only” approach — yet this can obstruct communication between children and parents and impede opportunities to provide the sexual education that has been demonstrated to reduce risky behavior (10). In African settings research has constantly shown that the influence of culture and tradition in parent-adolescent sexual communication. In Uganda, Tanzania, Zimbabwe and Ghana culture and tradition have been shown to act as a barrier of parents discussing sex with adolescents (2, 9, 11, 12). In such settings parents positioned adolescents as immature and irresponsible thus incapable of involving in sex. Where sex was discussed, for example in Uganda, it was used as a fear tactic — have sex and you will get HIV/AIDS (11). The use of fear has been seen to extend to issues such as teenage pregnancies (7). This use of fear becomes a barrier for adolescents to approach their parents to discuss about sexual matters. In all these examples of the use of fear, it can be seen that parents have a particular understanding of adolescence and sex is seen as threatening this view. Research from Zimbabwe has shown that the use of fear by parents arises from concerns that parents have about adolescence as a stage (3). Despite the difficulties in parents communicating about sexual matters with adolescence, it does not mean that adolescents are not engaging in sexual activities. In Zimbabwe, research has shown that adolescents have sexual agency which is not readily accepted by elders (1, 3, 13). In these situations adolescents are seen as social agents who are able to construct “their own sexual realities and identities” (13).

Parent-adolescent sexual relations have been variously theorised. In this paper our understanding of sexuality focuses on how adults perceive youth sexualities. The study brings to the fore the differences between the perceived reality of adults versus the lived realities of young people. We thus use the concept of ‘adolescent sexual denial’ to understand how elders perceive and construct adolescent sexualities. This concept is theoretically grounded in how historical processes have influenced the sexual scripts in Zimbabwe. In their 1973 book *Sexual Conduct* Gagnon and Simon define sexual scripts as what leads up to sexual encounters in which are learned interactions (14). Sexual scripts are “not primarily comprised of behaviours; rather, they are cognitive frameworks” that include norms that guide behaviour, individuals’ interpretations of the implications of cultural norms for interpersonal interactions, and individuals’ constructions of their own desires (15-16). As such they might be thought of as “both social agents, prescribing what is considered normative within a culture, and as intrapsychic maps, providing directions for how to feel, think, and behave in particular situations” (17). Parents and other adults communicate these sexual scripts as the assumed protectors of culture. The sexual scripts provide “individual actors with instruction as to the appropriate times, places, sequences, and so forth with regard to sexual activity” (17). Sexual scripts help understand how power influences the social construction of dominant ideas about sexuality and sexual norms. Following from Foucault we see these sexual scripts as knowledge systems that act through power relations which arrange sexuality in order to control sexual practices (18). In African settings patriarchal power relations mean the sexual scripts are thus gendered and aged. An example of the gendered nature of these scripts is seen in how male adolescents are given more leeway than female adolescents in sexual issues (3, 13). Patriarchy is central in African cultures, especially in relation to sexuality (19). In such settings patriarchal power relations are seen as creating and sustaining gender hierarchies in African societies by controlling sexuality and reproductive decision-making for example adolescent sexualities (19).

The sexual scripts which define sexuality for young men and women are understood within a framework were adolescents is seen as a stage of development that falls somewhere between childhood and adulthood. Sexual activities are understood as constructed from the interplay between cultural messages about sexuality, identification of situations as sexual, and interpersonal negotiation (20). These scripts have evolved in Zimbabwe and it is largely accepted now that adolescent sex is discouraged and young people are expected to abstain until marriage. This is partly based on cultural and religious believes. These cultural and religious beliefs form the basis of sexual scripts that guide behaviour of adolescents in terms of sexual activities. Zimbabwe is largely a Christian nation thus religion plays an important part in defining sexuality. Christianity defines pre marital sex as immoral and sinful. As such Christians are expected to be virtuous protecting their bodies that are the temples of the Lord. Adolescent sexual denial is based on such thinking. Traditional and cultural scripts also construct sex as an adult activity. Young people are supposed to grow up abstaining from sex and thus there is little need to discuss with them what sex is about. Parents believe that their children should be following these scripts yet evidence shows that they are not (13). In relation to Christianity and traditional values modern discourses on the dangers of adolescent sexualities have also been to exist in Zimbabwe. Here adolescent sexual activities are seen as risky, dangerous and harmful for future life prospects (3). Research has shown that the availability of cultural sexual scripts, traditional sexual scripts, danger sexual scripts and gendered sexual scripts does not necessary mean that they influence individual decisions (21). 

The question then becomes why parents continuing believing that their children are not sexually active? The answer can be seen in the interaction between the traditional and cultural meanings of parenting and the social construction of adolescence. As shown earlier adolescence is constructed as a stage between childhood and adulthood and as such requires careful navigation with the help of those who have gone through it before – parents and adults. Parenting in Zimbabwe is seen as a form of guarding and protecting adolescent children as they go through a dangerous phase in life. Due to this denial of adolescent sexualities becomes a powerful concept based on the inability to accept the truth about their children. This denial is only made in regards to their children. There is a contradiction in how generally parents believe that youths nowadays are loose yet deny that their own children are part of this highly sexual youth. This fear of highly sexualised youth reinforces the idea that parents have on the dangers of adolescence and acts as barriers in Zimbabwean parents communicating with the children on sex matters. It is the ‘not my child’ syndrome on which adolescent sexual denial is based on. What is important to note is that these scripts are negotiated in interaction. Sexual scripts are just templates that an individual can follow or refuse. Parents tend to ignore how their own children, despite how much warning and threats about sex will still built up their own knowledge and agency.

## Materials and methods

This paper is based on an exploratory research that was qualitative in nature seeking a grounded understanding of how parents make sense of adolescent sexuality. A total of ten in depth interviews, four focus groups and six key informant interviews with purposively sampled male and female respondents were conducted. Key informants included a headmaster, teacher, social worker, nurses and member of traditional healers association. Among the in depth interviews we focused on parents including single women with adolescent children of any sex. The methodology was designed to ensure that experiences and thoughts of adults on young people having sex are fully captured. Through allowing research participants to speak we were able to provide a nuanced analysis of how parents create perception about their children when it comes to sex. The study was augmented by review of literature on how adults have in the past dealt with adolescent sexuality. 

The study was conducted in Mbare, one of the oldest suburbs in Harare that is the capital of Zimbabwe. It was the first high-density suburb (township), being established in 1907. At that time, it was located near the city cemetery, sewage works, and abattoir. It was originally called Harare (Hariri) Township, a name later on used for the capital city itself. Mbare is the hub of informal economic activities with the biggest vegetable, arts and second hand clothes markets. The suburb has semi detached housing in an area called National and a number of flats which are in seriously bad states. This research mainly concentrated on parents living in the flats. The flats are plagued with sewage problems and lack of basic hygiene. The majority of the flats were constructed under colonization to cater for African labour force. They were meant mainly for single men with the belief that wives would stay in the rural areas. Now families are living in two roomed flats and in some cases two families sharing a flat. The context of the research is thus poor and overcrowded flats in Harare’s oldest suburb.

## Results and discussion

One thing stood out from our findings. Parents that were interviewed denied that their adolescent children were sexually active. This denial of adolescent sexuality was seen throughout the interviews. The denial of adolescent sexuality was linked to the other themes that emerged including sexual surveillance and sexual communication, school pregnancy, STIs and sexual education, and adult anxiety on adolescent sex. The extracts presented in the analysis below exemplify patterns that emerged in the data.

***‘Not my child’: Adults adolescent sexuality denial: ***Parents in Mbare showed an overwhelming interest in adolescent sex highlighting how pregnancies and sexually transmitted diseases were increasing among the youth in the area. One elderly female parent noted: 

*Long ago it used to be our children buried us but now we are burying them. There is so much sexual activity among young people. The young girls and boys no longer have morals. They cannot wait for marriage anyone. It is sad how many of them are having children as teenagers.*

 This view was share with many of the elders who indicated how having sex has become commonplace amongst young people. A social worker in the area noted similarly that rate of teenage pregnancies is increasing showing that adolescent girls are involved in unprotected sex. A community leader argued that, ‘*We have to accept that our children are increasingly having sex at a young age.*’ This was widely accepted by research participants in both interviews and focus group discussions. Early sexual debut and unprotected sex were seen as the biggest problems concerning the youth in the area. Parents and elders all seem to believe that sexual activity of adolescents is misbehaviour and immoral. They believe that sex is for adults and married people. According to the nurse interviewed:

*Sex is not for children. We see this young girls coming to the clinic with sexually transmitted diseases and pregnancies. It is sad because the men responsible are out of sight. We help them as much as we can but as a nurse your help is limited to the medical issues.*

This belief guides the practice of denying young education to children both at school and at home. Parents believe that talking about sex with children will increase their chance of becoming sexually active. One parent noted that: ‘*If you start teaching children about sex then it will be your fault if they start experimenting and using that knowledge.*’ Denying information about sex to adolescents is thus seen as a measure of ensuring that they do not become sexually active, the believe being that they will not practice what they have not been taught. Adolescents are not expected to know or be involved in sexual activity. A traditional healer noted that: 

*Sex is such an important part of African cultures. It is sacred because it is the link we have between the past, present and future. The past is that we are products of sex; the present in that we engage in sex to create the future which is our children. As such it is for those people who are mature and have been taught about what it means*.

Parents in the focus group discussions were mainly Christian, arguing that premarital sex is immoral. They argued that fornication is a sin and they teach their children not to sin against God. In such a context, adolescents are starved of information about sex in their homes. 

When asked about their own children, most parents refused to admit their children are part of youth engaging in illicit sexual behaviours. As most parents put it, ‘Not my child.’ One of the parents interviewed noted: ‘*I raised my child very well with manners and morals. I monitor all she does.*’ Interviews with headmaster and teacher brought out how parents are in denial of what their own children are doing. The headmaster argued that it was only public denial but privately they know their children are sexually active. He noted that: 

*Parents know what their children are doing. It is public shame and stigma that makes them deny it in public. They are afraid that people will judge them as bad parents. There are however some who genuinely do not know their children are sexually active. The children nowadays with all this technology are having different lives that their parents are not even aware of.*

Parents in in-depth interviews however reiterated that their children are not sexually active. Questioned further the parents agreed that they might have suspicions but they are strict with their children by giving them curfews, monitoring their movements and constantly warning them about pregnancy and HIV. One of the parents indicated that: 

*It is not possible to talk about sex openly with our children however we give them warnings about AIDS and pregnancy. It is difficult for a parent to accept that their children are’ immoral.*

Parents in the study showed an impressive level of denial. Adolescents’ actual behaviours are totally divorced from their parents’ view of what they really do. Most parents forget that teenagers are mostly young and sexually curious. Parents tend to see their own children as asexual despite evidence of increased teen sex. What is interesting is how adults seem to think sex is not good for children. In the focus group discussions participants’ portrayed adult sex as inherently good and teen sex inherently bad: ‘*Children have no business being involved with sex. It is adult business. They should wait for their time.*’ What is interesting however is the view that most parents have of other people’s children. It is always the neighbour’s child or a friend’s child who is naughty and sexually active. Parents tend to have a negative perception of other people’s children. A female participant noted that: ‘*many parents nowadays are lenient with their children. You see the girls with boys at odd hours yet the parents do not do anything. These kids are doing what they want.*’ It is always other peoples’ adolescents that are problematic and sexually active. Another male participant said that. ‘*Kids today are highly sexually active. They are always looking for sex. This is why I am always watching my children.*’ Seeking sexual pleasure by adolescents is viewed in a negative way and adults think that when they are grown they will know about sexual pleasure and desire. Denial about adolescent sexuality thus is only for one’s children. Other children maybe highly sexual and the age of sexual debut maybe going up but parents are adamant that their children are not involved. 

***Sexual surveillance and sexual communication: ***Sexual surveillance amongst parents is very high yet there is very little sex communication between adolescence and parents. Monitoring of children is done through knowing where they are all the time and gossip from other parents and youths. It is however the lack of communication about sex which is disconcerting. Adolescence can be a confusing period for children especially if no information is forthcoming. It coincides with emergence of a sexually maturing body and development of sexual desires. Adolescence is a time for youths to learn to understand and deal with sexual desires whilst experimenting with sexual behaviours. When asked about this lack of information, a male parent answered that:

*How do you start talking to your daughter or son about sex? They will lose respect for you. Children need to respect their parents. We are not friends if I know you are engaging in sex then you will be punished.*

This surveillance and punishment system makes it impossible for children to discuss sex with their parents. Parents have devised elaborate surveillance of adolescent sexualities. This includes curfews, monitoring of movements and monitoring all communications. In some cases this includes monitoring dressing and friends. As one parent puts it:

*You need to be vigilant especially with girls. I monitor everything including dressing to ensure they are not getting into the wrong things. I have timed all the distances from school and shops so they know that they will get punished if they come late from school or shops. Everyone is in the house by five including my boys and no one is allowed outside except when we have evening church services.*

The sexual surveillance tends to be gendered in multiple ways. The controls of movement seem to mainly target girls and the parents gave various reasons that are in many ways characteristic of partriachial societies. One reason is that it is girls who fall pregnant thus the need to monitor and protect them from this. Once the girl gets pregnant, she brings shame to the whole family. Another reason was that girls are easily swayed and influenced by friends and peers thus the need to ensure tight monitoring. 

This punishment is gendered in many ways. Girls are usually kicked out of the house and send to the house of the boy/man who they are sleeping with. Boys do not in most cases meet the same fate though due to economic hardships most parents noted that even sons who have girls eloping are forced out of the house. Parents who participated in this study noted that they were poor communicators about sex with their children. Women however noted how it was better with their daughters as they could tell them about sex as highlighted by one lady, ‘*With girls it is easier to discuss about sex and its implications. You warn them about early pregnancy and AIDS*.’ Men also seemed to believe that their wives talk regularly about sex with their daughters. This is based on a partriachial believe that when young girls get pregnant out of wedlock, it is the mother’s fault. In an interview one of the male parents argued that ‘*Obviously when a girl gets pregnant it is the mother’s fault. Mothers what their daughters are doing are up.*’ Asked whether fathers are in turn responsible for sons that impregnate girls out of wedlock, he said that, ‘*Boys are very secretive and it is difficult to know what they are doing plus boys will be boys.*’ This highlights gendered believes about young people’s relationship to their parents. Girls are generally stigmatised when they fall pregnant outside wedlock and their mothers are largely blamed. In the focus group discussions it was clear that most women agreed that the mother is to blame when a girl is impregnated. One woman actually noted:

*You get a bad name as a mother if your child gets impregnated out of wedlock. Women will start gossiping behind your back and many bad things are said about you. It is even worse if you have a position in the church but with the increase pregnancies it is getting better. Now women understand that there is little you can do to control your children.*

Cultural and societal expectations tend to exonerate husbands because women are seen as responsible for raising and instructing girl children. 

***School pregnancy, STIs and sexual education: ***Given the unanimous agreement by parents that young people are increasingly engaging in sex the participants where asked how they felt about teaching about and distributing condoms in schools. Adolescents usually engage in unsafe sexual practises because of a variety of reasons, which include lack of knowledge on proper, us of protection, lack of access to protection and for girls the lack of power to negotiate for condom use. To ensure that they are engaging in safe sexual behaviours knowledge of proper use of condoms is vital. The government is implicit in adolescence sexual surveillance by punishing pregnant school going girls. In August 2010, the Government of Zimbabwe amended the disciplinary code by granting maternity leave of up to three months, instead of automatic expulsion, for girls who fell pregnant during their school career. This decision was rescinded when parents and conservative groups including traditional leaders complained that this would promote promiscuity in schools. According to Circular No. 35 of 8 October, 1999, pregnancy of a learner, and being responsible for it, were misdemeanours of a serious nature. Schoolboys guilty of making their female counterparts pregnant would only be considered for readmission at another school after one year, as a sanction for misconduct. Parents who participated in this research were all against distribution of condoms and allowing pregnant girls to continue with schooling. One female parent argued that: 

*It is not good to have condoms in schools or to allow pregnant girls with other children. This will only promote children to engage in sex.*’ Another parent noted: ‘*If we allow condoms in schools what else must we allow. After this we will be saying let’s give them time and space to engage in sex without hiding to make it safer.*

 With or without condoms in schools children are having sex but parents are unwilling to accept this reality and offer them options to practice it safely. In the focus group discussions the parents showed stigma against pregnant girls in school. They called the girls immoral, loose and troublemakers who should not be around other children yet they thought boys who impregnate girls should remain in school. They argued that the boy has to continue with education so as to be able to care for the new family. Asked whether any of the parents had children who had fallen pregnant at school, six said yes. All the six agreed that pregnant girls should not be allowed in school, as one of them noted: ‘*It sends a bad example to other children who may think it is okay to fall pregnant.*’ 

When the government changed the law to allow pregnant girls there was widespread debate. Child protection activists, such as award-winning novelist Petina Gappah was quoted in newspaper reports hailing the introduction of maternity leave as progressive, noting that: ‘It is excellent news. As we all know, teens have sex, and sometimes the girl falls pregnant. It never made sense that in Zimbabwe, the girl who fell pregnant was expelled while the boy who made her pregnant remained in school to finish his education’ (http://www.newzimbabwe.com/news-3022-Maternity%20leave%20for%20school%20girls/news.aspx).

Punishment for being sexually active for young girls in Zimbabwe is not in any corrective. It punishes young women who suffer stigma at different levels and their lives are transformed for good. Current laws punish not only the mother, but also the unborn child. It has been noted that female students have their own needs that must be accommodated, which, if not acknowledged at policy formulation level, might not be addressed at policy implementation level (Chirimuuta, C. 2006 Retrieved from http://www.quietmoxmtainessavs.org). The right to education for all children, regardless of their condition, should be enshrined in national laws. The late sociologist and traditional healer Godwin Chavhunduka however disputed and cautioned that the education ministry would have to "send a clear message that this policy is not giving school children the licence to make children at will, otherwise the education sector will be thrown into confusion (http://www.newzimbabwe.com/news3022Maternity%20leave%20for%20school%20girls/news.aspx). Another conservative group called *Tsika Dzedu* (Our Culture) claimed that it is taboo and unmentionable in African to allow unmarried girls to get pregnant, let alone promote it. All these debates deny adolescent sexual agency. They are based on a socially constructed belief that sex is only for adults. As such adolescent sex has to be suppressed, as it is dangerous for society. 

***Adult anxiety on adolescent sex: ***Adolescent sexuality causes serious anxiety in adults. Many parents do not want to see their children as sexual beings. This asexualisation of children partly explains why discussions about sex within families remain a taboo. Sex is a controversial and contested issue in that parents would want to keep their children far away from such talk. What is problematic is however is the emergence of new media and sexual images being saturated across all spaces. Even cartoons have scenes of kissing with mild but still powerful sexual innuendos. It is thus surprising to note the anxiety in most adults in Zimbabwe when openly discussing about sex. One parent argued that:

*Growing up sex was never a subject you discussed with your father. Even with your mother the discussions were about abstinence and pregnancy. We were never told about pleasure or of sex as fun. Our parents showed us that in our culture sex is private. Only girls who are about to be married are told secret and intimate details by their aunts.*

The majority of parents are not equipped to deal with the sexual realities of their children thus they bury their heads in the sand as a coping mechanism. In a key informant interview, a social worker noted that:

*In the vast majority of homes it is difficult to broach topic on sex. It is a taboo. Mentioning the word causes panic yet it is a reality we leave with everyday. Parents would want to believe their children are naive yet the truth is most children know about sex*.

Even at the national level any debates about sex tend to raise a public moral panic followed by an outcry about moral decadency. It is curious why sex causes such a reaction from the people who are practicing it. With fears for pregnancy and diseases, most parents are not comfortable with their children being sexually active. Some parents are of the view that talking about sex to young people might encourage them to want to experiment and see how it feels. Parents construct their children as little kids who have no interest in sex. They are afraid of accepting that their adolescents are sexual beings. As Elliot noted in an interview:

We’ve done a really good job of constructing sexually active teens in a highly negative way, in a way that really emphasizes the risks, the peril — and not the pleasure. When’s the last time you heard anything about teen sexual activity that was positive? Parents aren’t hearing anything positive about teen sexuality, they’re hearing about the profound physical, psychological and social consequences of sex. So of course when they look at their teens they don’t want to imagine their kid encountering those risks (http://www.salon.com/2012/08/12/sex_not_my_kid/)

This view was well articulated by most of the research participants who just refused to admit their own children are sexually active. They noted that they were ‘good’ and strict parents thus they are sure their children are not sexually active.

## Conclusion

The foregoing discussion has shown how parents construct the sexuality of their adolescent children. Parents tend to have positive bias about their children and thus are unwilling to accept that their children can be sexuality active. Admitting that your child is sexually active is seen as admitting to being a bad parent who has failed in controlling their kids. The irony is that the parents perceive that adolescents are increasingly becoming sexually active. Findings of the study highlight how communication about sex remains a complex and difficult undertaking in Zimbabwe.
